# Multimodal Mobility Assessment Predicts Fall Frequency and Severity in Cerebellar Ataxia

**DOI:** 10.1007/s12311-021-01365-1

**Published:** 2022-02-04

**Authors:** Roman Schniepp, Anna Huppert, Julian Decker, Fabian Schenkel, Marianne Dieterich, Thomas Brandt, Max Wuehr

**Affiliations:** 1 Department of Neurology, University of Munich, Marchioninistrasse 15, 81377 Munich, Germany; 2 German Center for Vertigo and Balance Disorders (DSGZ), University of Munich, 81377 Munich, Germany; 3 Schön Klinik Bad Aibling, Bad Aibling, Germany

**Keywords:** Cerebellar ataxia, Falls, Fall prediction, Gait analysis, Mobility monitoring

## Abstract

This cohort study aims to evaluate the predictive validity of multimodal clinical assessment and quantitative measures of in- and off-laboratory mobility for fall-risk estimation in patients with cerebellar ataxia (CA).

Occurrence, severity, and consequences of falling were prospectively assessed for 6 months in 93 patients with hereditary (*N* = 36) and sporadic or secondary (*N* = 57) forms of CA and 63 healthy controls. Participants completed a multimodal clinical and functional fall risk assessment, in-laboratory gait examination, and a 2-week inertial sensor-based daily mobility monitoring. Multivariate logistic regression analyses were performed to evaluate the predictive capacity of all clinical and in- and off-laboratory mobility measures with respect to fall (1) status (non-faller vs. faller), (2) frequency (occasional vs. frequent falls), and (3) severity (benign vs. injurious fall) of patients.

64% of patients experienced one or recurrent falls and 65% of these severe fall-related injuries during prospective assessment. Mobility impairments in patients corresponded to a mild-to-moderate ataxic gait disorder. Patients’ fall status and frequency could be reliably predicted (78% and 81% accuracy, respectively), primarily based on their retrospective fall status. Clinical scoring of ataxic symptoms and in- and off-laboratory gait and mobility measures improved classification and provided unique information for the prediction of fall severity (84% accuracy).

These results encourage a stepwise approach for fall risk assessment in patients with CA: fall history-taking readily and reliably informs the clinician about patients’ general fall risk. Clinical scoring and instrument-based mobility measures provide further in-depth information on the risk of recurrent and injurious falling.

## Introduction

Gait disturbances are a common and disabling complication in patients with cerebellar ataxia (CA) with significant implications for patients’ capacity for independent living and quality of life [1, 2]. Walking impairments in CA are linked to a considerable risk of recurrent falling, which frequently results in severe secondary comorbidities and a loss of functional independence. Retrospective and prospective fall surveys in CA indicate that 74–93% of patients experience at least one fall per year [3–5]. Many of these patients suffer from fall-related injuries (74–85%), which potentially (23–31%) result in a fracture or joint dislocation [3, 4]. Since causal therapeutic approaches are currently not available [6, 7], a primary treatment objective in CA is an effective prevention of falling and fall-related injuries.

Fall prevention in CA requires reliable screening and classification procedures to assess and identify individuals at particular risk of falling and severe fall-related complications. Distinct risk factors including symptom severity, the number of non-ataxic symptoms, and the duration and etiology of disease were associated to higher fall rates in CA patients [3–5, 8]. However, the predictive validity of these factors considerably differed between studies. Complementary approaches aim to identify fall risk-related features linked to balance and gait impairments in CA. Accordingly, variability in the movement pattern, a defining feature of gait ataxia [9, 10] that increases with disease severity [11], was shown to be associated with patients’ general fall status and risk of recurrent falling [2, 11, 12]. Subsequent analyses, however, indicated that gait variability markers may be only predictive for a specific subclass of falls (falls resulting from tripping or stumbling) [13]. Furthermore, quantitative gait assessment is currently mostly restricted to in-laboratory settings and thus potentially underestimates the challenges of everyday-life mobility during which patients actually fall.

Recent advances in off-laboratory mobility assessment using body-worn inertial sensors promise a more specific characterization of patients’ balance impairments and fall risk in environmentally valid settings [14]. Inertial sensor-based mobility assessment has been shown to consistently capture central features of ataxic gait impairments under real-life conditions [15]. In contrast to in-laboratory gait assessment approaches that primarily focus on a detailed characterization of spatiotemporal features of walking impairments, body-worn sensors may further provide important complementary insights into patients’ overall mobility status and balance capabilities from a macroscopic perspective [14, 16–19]. This information could supplement and overcome limitations of currently available screening tools for fall risk assessment in CA.

The aim of this study was to evaluate and compare the predictive validity of currently available and recently established screening tools for fall risk identification in patients with CA. To this end, we prospectively assessed the occurrence, frequency, and consequences of falling in a comprehensive cohort of patients with CA of different etiologies. Subsequently, multimodal score-based and clinical fall risk assessment outcomes as well as measures from instrument-based in-laboratory gait examination and long-term off-laboratory mobility monitoring were evaluated with respect to their relevance for forecasting the occurrence, frequency, and severity of falls.

## Methods

### Participants

Longitudinal cohort study with 93 patients with CA and 63 healthy controls recruited (study ID: DRKS00007762) from 06/2015 to 02/2018 at the University Hospital Munich. Patients were included based on the following criteria: (1) presence of clinical ataxia [20] due to sporadic, secondary, or hereditary forms of CA, (2) age between 18 and 80, and (3) ability to ambulate independently without walking aids. Inclusion criteria for controls were (1) no history of neurological or psychiatric disease, (2) age between 18 and 80, and (3) no other diseases with manifest ambulation problems.

### Clinical Work-Up and Fall Assessment at Inclusion

All patients and controls underwent a standardized physical examination, which included a survey of the following information: ambulatory status, functional status, medication, and falls within the preceding 6 months. According to the WHO criteria, a fall was defined as an event which results in a person coming to rest inadvertently on the ground or floor or other lower level [21]. A near-fall was defined as an event that results in a marked postural instability requiring a supporting step and/or balance adjustment. Retrospective fall assessment included information on fall status (faller vs. non-faller), fall frequency (no, occasional, frequent falls (≥ two falls)), and fall severity (1: near-fall; 2: no or mild injury; 3: injury requiring medical attention; 4: injury requiring hospital admission) according to the Hopkins Falls Grading Scale (HFGS) [22]. The subjective level of stability was assessed by the Falls Efficacy Scale-International (FES-I) and the Activities-specific Balance Confidence Scale (ABC-d) [23, 24]. Health-related quality of life was assessed by the Short-Form Health Survey (SF-12) [25]. Cognitive function was screened with the Montreal Cognitive Assessment (MOCA) [26]. Each participant underwent a complete neurological and physical examination including the assessment of functional mobility by the Timed up and Go Test (TUG) and the Functional Gait Assessment Score (FGA) [27, 28]. Severity of ataxia symptoms in patients was rated using the Scale for the Assessment and Rating of Ataxia (SARA) with a maximum of 40 points (indicating most severe form of ataxia) [29].

### Prospective Fall Assessment

Each participant was provided with a fall diary covering a 6-month follow-up period. Participants were asked to document near-fall and fall events on a daily basis with information on the time, the environmental circumstances, the fall mechanism (e.g., tripping, vertigo/dizziness, impaired consciousness, others), the duration of the post-fall lying phase, and the related HFGS of each event. Participants were additionally contacted by phone monthly to cross-check and validate the documented information. Based on the prospective fall assessment, participants were categorized with respect to fall status, frequency, and severity.

### In-laboratory Gait Examination

In-laboratory gait assessment was performed on a 6.7 m-long pressure-sensitive carpet (GAITRite®, CIR System, Sparta, NJ, USA) at 120 Hz. Participants walked over the carpet at their self-chosen (PWS), slow (SWS), and maximum walking speed (MWS). Each condition was recorded 4 times. Gait assessment was conducted without additional ambulatory aids. For each walking condition, the following spatiotemporal gait parameters were analyzed: gait velocity, base of support, stride length, stride time, swing phase percentage, double support percentage, coefficient of variation (CV) of the base of support, CV of stride time, CV of stride length, gait asymmetry index, and phase synchronization index [30].

### Off-laboratory Mobility Assessment

Following the initial visit, monitoring of daily mobility was undertaken for 14 days. Participants wore an inertial sensor-based activity monitor (ActivPAL®, PAL Technologies, Glasgow), which recorded the sequence and period of individual bouts of ambulatory, sedentary, and sleeping behavior at 10 Hz. The inertial sensor was placed on the thigh of the dominant leg approximately 0.1 m cranially and 0.05 m laterally to the patella. Participants were advised to continue their daily activities as usual and not to change their routine. Upon completion of the recording period, participants removed the sensor by themselves and send it back by mail.

The following parameters (expressed as average daily estimates) were computed from the ActivPAL data [31, 32]: daily intensity (amount of daily energy expenditure expressed as the total metabolic equivalents (METS)), daily volume (percentage of ambulatory, sedentary, or sleeping time), daily step count, daily number of sit-to-stance transitions, daily pattern of ambulatory behavior (computed as the exponent alpha that quantifies the distribution of bouts, with lower alpha values indicating a greater contribution of long bouts).

### Data Analysis Procedures

Descriptive statistics are reported as mean ± SD. In a first step, analysis of variance (ANOVA) and chi-squared tests were used to test for differences of metric and categorical parameters from clinical assessment, in-laboratory gait examination, off-laboratory mobility assessment, and retro- and prospective fall assessment between patients and controls. The subsequent statistical analyses focused solely on patient data: multivariate backward logistic regression analyses (controlled for age, gender, and leg length) were performed to identify independent predictors associated with the three dependent prospectively assessed fall measures of interest: (I) non-faller vs. faller, (II) occasional vs. frequent faller, and (III) non-severe vs. severe falling (defined as HFGS 3 or 4). For each regression model sensitivity, specificity and correct classification are reported as quality parameter derived by the classification matrix. Initially, all potential predictor parameters from clinical and mobility assessment were subjected to an ANOVA (including post hoc comparison via Sheffé procedures) with respect to the three above-mentioned fall categories. Subsequently, only those parameters that yielded a significance level of their *F* value ≤ 0.05 were considered in the respective regression model. To avoid collinearity, relationships among parameters were examined using Pearson’s correlations. If parameters were strongly correlated (*r* > 0.7), only the one most strongly associated with the dependent measure was retained. This statistical approach is based on [33]. Statistical analysis was performed using SPSS (Version 25.0; IBM Corp., Armonk, NY).

## Results

### Characteristics of the Study Cohort

Demographic information and clinical characteristics of patients and controls are summarized in Table 1. The patient cohort included hereditary degenerative forms of ataxia (*N* = 36), sporadic degenerative forms of ataxia (sporadic adult-onset ataxia, *N* = 36), ataxia due to focal vascular or neo-plastic cerebellar lesions (*N* = 3), and downbeat nystagmus syndrome (*N* = 18). Patients had a mean SARA score of 10.2 (range: [3;24]), indicating an on average moderate severity of ataxic symptoms. Accordingly, assessment of functional mobility revealed mildly impaired balance and gait capacity of patients compared to controls (FGA: *F*_1,153_ = 91.4, *p* < 0.001) despite normal performance in the TUG. Patients reported increased fear of falling (FES-I: *F*_1,153_ = 78.5, *p* < 0.001), decreased balance confidence (ABC-d: *F*_1,153_ = 114.8, *p* < 0.001), and showed moderately reduced cognitive function (MoCA: *F*_1,153_ = 16.3, *p* < 0.001). Health-related quality of life scores did not differ between patients and controls.

**Table 1:** Characteristics of the study cohort

	Healthy subjects	Cerebellar disorders		*F*_1,153_			*p*
**Demographical characteristics**							
*N* (f/m)	63 (31/32)	93 (35/58)					
Age [y]	49 ± 14	57 ± 18		2.4			n.s
Diagnoses		14 SCA 11 FRDA 11 EA2 18 DBN 35 SAOA 1 ARAC 1 ACM 1 post stroke					
**Clinical performance scales**							
SARA score [points]	–	10.2 ± 2.6					
FGA [points]	29 ± 3	21 ± 6		91.4			< 0.001
TUG [s]	8.8 ± 3.2	11.2 ± 7.1		0.7			n.s
MOCA [points]	29 ± 3	24 ± 6		16.5			< 0.001
**Subjective symptome scales**							
ABC-d [%]	95 ± 9	63 ± 22		114.8			< 0.001
FES-I [points]	17 ± 2	31 ± 13		78.5			< 0.001
SF-12 [points]	31 ± 3	30 ± 4		1.15			< 0.001
				df	c^**2**^		*p*
**Retrospective falls status**							
No falls [*n*, %]	57, 90	32, 34		2	37.2		< 0.001
Occasional fall [*n*, %]	4, 7	21, 23					
Frequent falls [*n*, %]	2, 3	40, 43					
**Retrospective falls severity**							
Hopkins grade I [%]	28, 45	22, 24		3	46.8		< 0.001
II [%]	17, 27	46, 49					
III [%]	17, 27	14, 15					
IV [%]	0, 0	11, 12					

*f*, female; *m*, male; *FGA*, functional gait assessment; *FES-I*, falls efficacy scale – international; *TUG*, timed-up-and-go test; *MOCA*, montreal cognitive assessment; *SF-12*, short form 12; *BVP*, bilateral vestibular failure; *UVP*, unilateral vestibular failure; *SCA*, spinocerebellar ataxia; *FRDA*, friedreich ataxia; *EA2*, episodic ataxia type 2; *DBN*, downbeat nystagmus syndrome; *SAOA*, sporadic adult onset ataxia; *ARAC*, autosomal recessive cerebellar ataxia; *ACM*, tumor cerebellar; C^2^, chi-square test

### In- and Off-laboratory Mobility Assessment

Descriptive information on outcomes from in- and off-laboratory mobility assessment is summarized in Table 2. During in-laboratory gait assessment, patients showed walking impairments corresponding to an ataxic gait disorder with reduced walking speed (*F*_1,153_ = 39.9, *p* < 0.001), prolonged double support phases (*F*_1,153_ = 22.6, *p* < 0.001), increased base of support (*F*_1,153_ = 65.7, *p* < 0.001), spatiotemporal variability (stride length *CV*: *F*_1,153_ = 21.3, *p* < 0.001; stride time *CV*: *F*_1,153_ = 28.1, *p* < 0.001), and asymmetry within their stride-to-stride pattern (gait asymmetry: *F*_1,153_ = 24.5, *p* < 0.001; phase synchronization: *F*_1,153_ = 17.3, *p* < 0.001). Off-laboratory monitoring of daily life mobility revealed a general reduction of energy expenditure (*F*_1,153_ = 48.7, *p* < 0.001) with a reduced daily amount of time spent during ambulation (*F*_1,153_ = 45.2, *p* < 0.001) and a smaller step count (*F*_1,153_ = 45.1, *p* < 0.001) in patients compared to controls.

**Table 2:** In- and off-laboratory gait and mobility assessment

	Healthy subjects	Cerebellar disorders	*F*_1,153_	*p*
**In-laboratory gait measures**				
Gait velocity [m/s]	1.2 ± 0.2	0.9 ± 0.2*	39.9	< 0.001
Stride length [m]	1.3 ± 0.2	1.1 ± 0.3	34.5	< 0.001
Stride time [s]	1.1 ± 0.1	1.3 ± 0.1*	9.0	0.003
Swing phase [%]	38 ± 2	37 ± 2	63.4	< 0.001
Double support phase [%]	24 ± 4	30 ± 9*	22.6	< 0.001
Base of support [m]	0.08 ± 0.03	0.15 ± 0.06*	65.7	< 0.001
Base of support CV [%]	24 ± 15	24 ± 12	0.7	n.s
Stride length CV [%]	2.2 ± 1.1	5.6 ± 4.6*	21.3	< 0.001
Stride time CV [%]	2.3 ± 1.9	5.2 ± 1.2*	28.1	< 0.001
Gait asymmetry index [%]	2.5 ± 1.5	6.8 ± 3.7*	24.5	< 0.001
Phase synchronization [%]	4.1 ± 1.4	9.9 ± 5.3*	17.3	< 0.001
**Mobility**				
*Adherence*				
Days recorded [d]	12.3 ± 1.9	12.4 ± 1.2	0.2	n.s
Time sensor worn [%]	96 ± 12	99 ± 6	0.8	n.s
*Volume*				
Median step count ± SD [#]	10,208 ± 3,299	6305 ± 3,302*	45.1	< 0.001
Mean ambulation % ± SD [%]	8.7 ± 2.6	5.7 ± 2.5*	45.2	< 0.001
Mean sedentary % ± SD [%]	27.8 ± 7.7	34.5 ± 10.2*	39.2	< 0.001
Median sleep % ± SD [%]	40.8 ± 6.7	42.3 ± 9.9	0.9	n.s
*Activity*				
Median ambulation bout # ± SD [#]	456 ± 134	338 ± 139*	23.8	< 0.001
Ambulatory bout duration ± SD [s]	17 ± 5	15 ± 4	9.8	.0.02
Mean daily intensity ± SD [MET]	35 ± 1	33 ± 1*	48.7	< 0.001
*Pattern*				
Mean ambulation alpha ± SD	1.42 ± 0.04	1.43 ± 0.04	1.5	n.s
Median sit-walk transitions ± SD	40 ± 16	36 ± 13	2.7	n.s

^*^indicates significant difference in the Sheffé posthoc comparison (to healthy subjects)

*CV*, coefficient of variation; *SD*, standard deviation

### Fall Assessment

Fall epidemiology in patients and controls is summarized in Tables 1 and 3. Both retrospective and prospective assessments revealed a considerably higher incidence of fallers, recurrent fallers, and severe fall-related injuries in patients compared to controls. According to the retrospective fall assessment, 66% of patients had experienced at least one fall within the last 6 months and 66% of those reported recurrent falling. Thirty-six percent of patients that fell reported fall-related injuries that required medical attention.

**Table 3:** Outcomes form prospective fall assessment

	Proportion of fallers				Fall events			
	Healthy subjects *N* = 56		Cerebellar disorders *N* = 80		Healthy subjects *N* = 9 falls		Cerebellar disorders *N* = 313	
	*N*	**%**	*N*	%	*N*	**%**	*N*	**%**
**Fall epidemiology**								
No falls	47	**84**	28	**36**	–	**–**	**–**	**–**
Occasional fall	9	**16**	18	**23**	9	**100**	18	**6**
Frequent falls	0	**0**	34	**41**	0	**0**	295	**94**
	**Chi**^**2**^** < 0.001**				**Chi**^**2**^** < 0.001**			
**Fall severity (HFGS)**					*N* = 18		*N* = 925	
1	4	**31**	10	**14**	9	**50**	612	**66**
2	5	**38**	44	**63**	5	**20**	293	**32**
3	4	**31**	14	**18**	4	**22**	16	**2**
4	0	**0**	4	**5**	0	**0**	4	**0**
	**Chi**^**2**^** < 0.001**				**Chi**^**2**^** < 0.001**			

Information on falls epidemiology on considers actual fall events (excluding near-fall events). Information on falls severity considers both fall and near-fall events

*HFGS*, Hopkins falls grading scale

In the 6-month follow-up period, all participants registered fall information in their falls diary that was counter-checked by structured monthly telephone interviews. Falls diary information from 13 patients and 7 controls was considered invalid and excluded from further analysis due to missing telephone contact data or discrepancies between the falls information documented in the diary and surveyed during monthly phone interviews. The total numbers of near-fall and fall events in patients registered during prospective assessment were 313 and 612, respectively. A majority of falls in patients with CA occurred indoors (75%) in domestic environments (71%), primarily on even surface (71%). Most falls were linked to walking or turning (71%) and 74% occurred either forwards or backwards. The most frequent causes of falling in patients were tripping (33%) and imbalance (23%). The most commonly reported associated symptoms with falling were dizziness and vertigo (20%) (Tables 3 and 4). A total of 5% of falls resulted in injuries which required outpatient medical treatment and 1% necessitated inpatient medical treatment. Overall, outcomes from prospective assessment revealed that 64% of the patients experienced falls within the 6-month follow-up period and 65% of these patients reported recurrent falling. Severe fall-related injuries that required medical attention occurred in 29% of patients that fell (Table 3).

**Table 4:** Prospective fall assessment. Fall event details

	fall events			
	Healthy subjects *n* = 9 falls		Cerebellar disorders *n* = 313	
	*n*	**%**	*n*	**%**
**Environment**				
At home	2	**22**	207	**71**
Public area	7	**78**	83	**29**
Indoor	2	**22**	218	**75**
Outdoor	7	**78**	72	**25**
	**xx**			
Even underground	0	**0**	210	**71**
Uneven underground	1	**11**	10	**3**
Stairs	1	**11**	28	**9**
Incline/decline	2	**22**	4	**1**
Slippery underground	3	**33**	22	**7**
Obstacle	2	**22**	21	**7**
**Circumstances**				
Morning	0	**0**	84	**28**
Noon/afternoon	4	**44**	144	**48**
Evening	5	**56**	68	**23**
Night	0	**0**	16	**5**
**Symptoms**				
Dizziness/ vertigo	0	**0**	60	**20**
Pain	2	**22**	5	**2**
Weakness	0	**0**	6	**2**
Collapse	0	**0**	14	**4**
Not specified	7	**78**	228	**73**
**Sequence**				
Anterior	5	**56**	101	**35**
Posterior	1	**11**	114	**39**
Lateral	3	**33**	69	**24**
Vertical	0	**0**	5	**2**
Tripping	2	**22**	104	**38**
Slipping	3	**33**	22	**8**
Weakness	0	**0**	46	**17**
Instability/ imbalance	0	**0**	88	**32**
Inattentive	0	**0**	9	**3**
Contact/ collision	4	**44**	3	**1**
**Behavior**				
Locomotion	4	**44**	203	**66**
Transition	0	**0**	42	**14**
Turning	2	**22**	16	**5**
Reaching/leaning	0	**0**	26	**9**
Standing	0	**0**	19	**6**
Sitting	0	**0**	1	**0**
Doing sports/ activities	3	**33**	2	**0**

### Multivariate Fall Classification Models

**Fig. 1:**
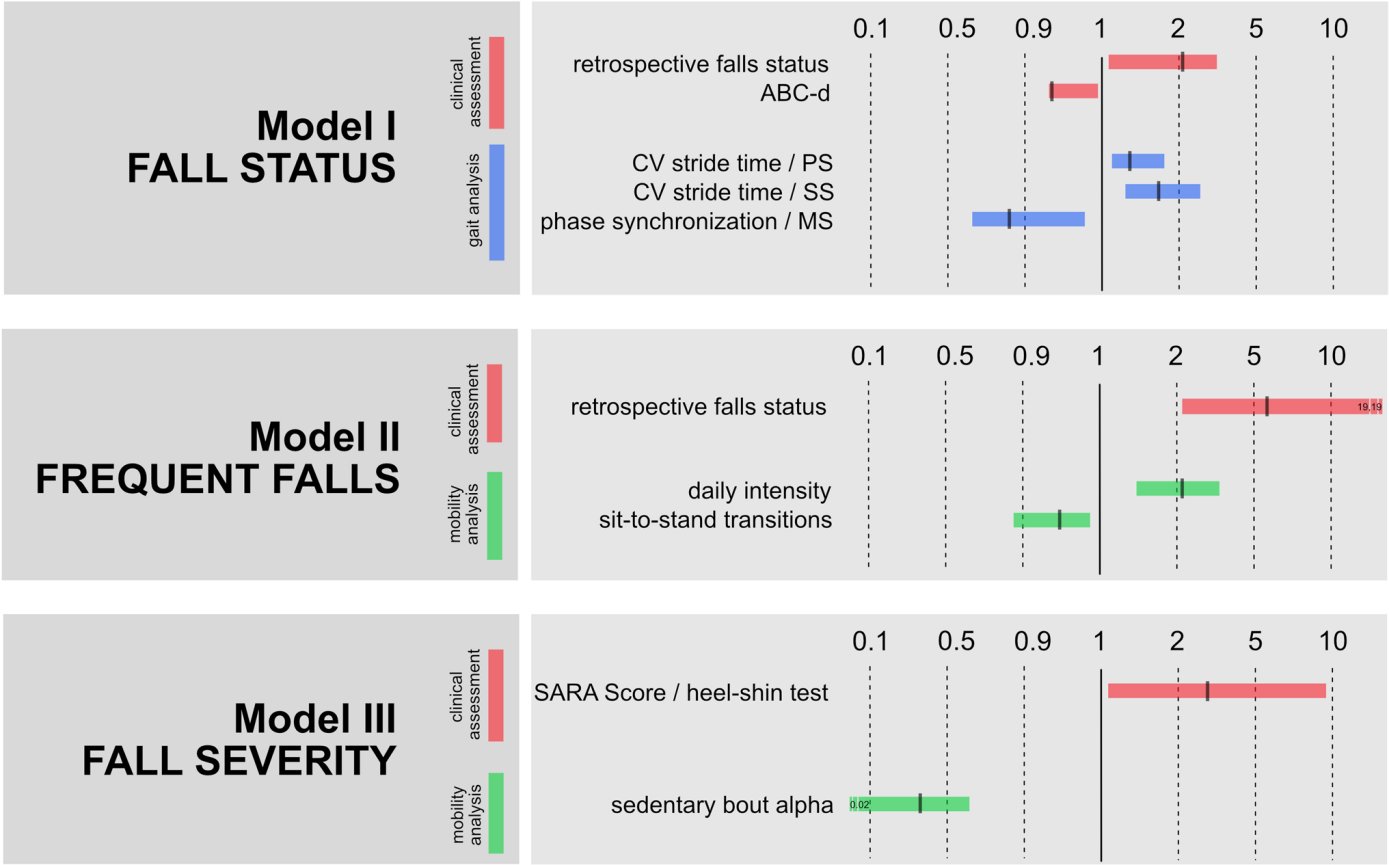
Overview of predictive factors for fall events in CA. Figure 1 represents the relevant factors for predicting fall events (model 1-3). Paramters were derived from clinical assessment (red), in-laboratory gait assessment (green), and off-laboratory mobility assessment (blue). Bars represent the limits of Exp(beta) of the regression models

The predictive model for fall status (non-faller vs. faller) was obtained after 10 iteration steps and yielded a correct prediction of 78% (sensitivity: 70%; specificity: 86%). The model included 5 predictive factors from socio-demographic and in-laboratory mobility assessment, with the most important being retrospective fall status and CV of stride time during slow walking (Table 5, Section A). Accordingly, a positive history of falls and impaired dynamic walking stability were the most important risk factors for experiencing falls during follow-up. No parameter of the mobility assessment was significantly represented in this model (Fig. 1).

The predictive model for fall frequency (occasional vs. frequent faller) was obtained after 13 iteration steps and achieved a correct prediction of 81% (sensitivity: 77%; specificity: 86%). The model included 3 predictive variables from socio-demographic and off-laboratory mobility assessment, with the most influential being retrospective fall status and intensity of daily mobility (Table 5, Section B). Thus, besides a positive history of falls, high daily activity levels were an independent risk factor for frequent falling during follow-up assessment (Fig. 1).

The predictive model for fall severity (falls that do vs. do not necessitate medical attention) was obtained after 12 iteration steps and yielded a correct prediction of 84% (sensitivity: 75%; specificity: 89%). The model only considered 2 parameters from clinical and off-laboratory mobility assessment, namely the subscore “heel-shin-test” of the SARA and ambulatory bout alpha (Table 5, Section C) (Fig. 1).

**Table 5:** Multivariate fall classification models

	Model information	Parameter information						
	Correct prediction	Coefficient	SE	df	*p*	Exp(b)	Low	High
A)**Model fall status**	0.78							
Retrospective fall status		0.73	0.32	1	0.024	**2.076**	1.101	3.913
ABC-d		− 0.03	0.02	1	0.037	**0.936**	0.940	0.998
Preferred walking—CV of stride time		0.25	0.16	1	0.045	**1.289**	1.102	1.761
Slow walking—CV of stride time		0.65	0.21	1	0.031	**1.757**	1.251	2.321
Maximally fast walking—phase synchronization index		− 0.22	0.10	1	0.022	**0.806**	0.670	0.971
B)**Model fall frequency**	0.81							
Retrospective fall status		1.92	0.53	1	0.001	**6.850**	2.445	19.193
Daily intensity		0.83	0.12	1	0.004	**2.382**	1.309	4.333
Sit-to-stand transitions		− 0.059	0.03	1	0.033	**0.943**	0.893	0.995
C)**Model fall severity**	0.84							
SARA subscore heel-shin test		1.16	0.56	1	0.037	**3.201**	1.075	9.534
Sedentary bout alpha		− 1.45	0.95	1	0.044	**0.467**	0.002	0.621

*ABC-d*, activity-specific balance confidence scale; *CV*, coefficient of variation; *SARA*, scale for the assessment and rating of ataxia

## Discussion

In this study, we prospectively assessed the occurrence, circumstances, and consequences of falling in patients with CA and evaluated the predictive validity of a comprehensive set of clinical and instrument-based health and mobility screening tools for identifying patients at particular risk of falling. Prospective fall assessment revealed that recurrent and injurious falling is a severe complication already in mild-to-moderate stages of cerebellar disease. Multimodal measures from clinical and in- and off-laboratory assessment not only allowed us to characterize complementary aspects of the health and mobility status in CA, but were differentially associated with distinct aspects of fall events in patients with CA. Consequently, multivariate fall risk classification models built on these measures yielded a high accuracy that outperforms previous models that only considered a limited set of explanatory characteristics. In the following, we will discuss the differential relationships between health status, mobility impairments, and risk of falling in CA and propose a guideline for a multi-level, stepwise approach for fall risk assessment in these patients.

### Balance and Mobility Impairments

Patients in our study cohort had different etiologies of CA at rather early stages of disease with mild-to-moderate symptom severity indicated by an average SARA score of 10 points. Clinical scoring of functional mobility in these patients demonstrated moderate impairments of balance and gait regulation. In-laboratory gait assessment showed corresponding walking characteristic of the staggering, broad-based phenotype of ataxic gait disorders [9, 10, 34, 35]. Balance and gait impairments both correlated with disease severity and were linked to reduced subjective balance confidence and increased fear of falling [11].

Off-laboratory measures of daily activity indicated only mildly affected everyday mobility behavior in patients with moderate reductions in the total volume and intensity of ambulatory activity. Patients performed less and shorter periods of walking and spent more time with sedentary behavior. In contrast to gait impairments, alterations of patients’ daily mobility were mainly independent of ataxia severity. Correspondingly, patients reported near-to-normal health-related quality of life scores (SF-12). In contrast, two previous studies on patients with hereditary CA at more advanced disease stages observed considerable impairments of daily activities that correlated with the severity of ataxic symptoms [18, 19]. Our findings thus suggest that despite apparent balance and gait impairments, patients at early stages of CA are more or less able to manage and retain near-to-normal daily life activity. However, impairments of daily mobility become increasingly relevant and disabling for patients at later stages of disease.

### Fall Epidemiology and Fall Risk Prediction in Patients with CA

Our 6-month prospective fall assessment in patients with CA revealed a fall incidence of 64%, which lies within the range of previous reports (50% in a 3-month assessment period [3], 84% in a 1-year assessment period [5]). Two third of patients that fell experienced recurrent falling and one third suffered from severe fall-related injuries requiring medical attention or even inpatient treatment. Furthermore, direct comparison of retrospective and prospective fall assessment outcomes revealed a high agreement with respect to fall status, frequency, and severity. Overall, retrospective and prospective fall assessment outcomes both emphasize that recurrent and injurious falling are frequent already in early stages of CA despite the relatively preserved ability for independent ambulation in these patients.

The relative distribution of circumstances and mechanisms of falling reported during prospective assessment largely confirm previous reports [3, 5, 36]. Most falls of patients occurred indoors on even ground and were linked to activities of walking or turning. The predominant causes of falling were tripping and postural instability. This suggests that most falls in CA are intrinsically generated in situations where the center of mass moves outside the base of support and dynamic balance cannot be adequately recovered. Furthermore, the high rate of sensations of vertigo and dizziness associated with falling in patients supports previous reports on the prevalence of persistent or episodic symptoms of vertigo and dizziness in CA (“cerebellar vertigo and dizziness”) [37, 38].

To identify explanatory variables that may predict patients’ fall status, frequency, and severity, we performed multiple regression analyses considering a wide range of sociodemographic characteristics and outcomes from clinical, self-report-based, and in- and off-laboratory gait and mobility assessments. Predictive models for each of the three fall categories yielded good-to-excellent classificatory performance (78–84%) with respect to previously established criteria for fall risk assessment approaches [39, 40]. Accurate prediction of both patients’ general fall status and fall frequency primarily relied on information from retrospective fall assessment. Accordingly, the presence of previous falls was the single most influential predictor for experiencing one or more falls in the 6-month follow-up assessment. Analogous to fall risk assessment guidelines in geriatric populations [41, 42], this finding suggests that patients’ fall history should be routinely surveyed during general medical history taking in order to readily identify those patients with particular risk of falling.

Prediction of patients’ general fall status further relied on parameters from in-laboratory gait assessment. In particular, increased levels of gait variability were identified as an independent risk factor for experiencing falls during the follow-up period. Irregularity of movement is a defining characteristic of cerebellar disease [9, 10] and scales with the severity of symptoms [11]. A close link between increased gait variability and the risk of falling in CA has been previously reported based on retrospective fall information [2, 11]. This relationship has been shown most prominent for slow walking modes. The current findings further complement this hypothesis by giving evidence for a relationship between gait variability at preferred speed walking and prospective fall risk in patients with CA. Gait irregularities have been particularly linked to the risk of falling due to tripping during undisturbed walking [36] — the most prevalent fall mechanism in our cohort. Thus, instrument-based measures of gait instability appear to be a suitable tool to improve the baseline fall risk estimation in patients with CA.

Interestingly, off-laboratory mobility measures did not contribute to basic fall risk estimation, but only became relevant for identifying patients at particular risk of frequent and injurious falling. Accordingly, prolonged periods of sedentary activity were predictive for injurious falling and a higher intensity of daily-life activity for experiencing recurrent falls. At first glance, the latter observation appears to be counterintuitive, since the level of daily physical activity is considered a global health marker [43] and training-induced increases in physical activity were found to not affect the risk of falling in an elderly population [44]. However, previous reports correspondingly observed that higher amounts of physical activity are associated to an increased fall risk in the elderly population [45] and patients with early Parkinson’s disease [17]. This suggests that especially patients with early-stage gait impairments who still maintain near-to-normal levels of daily activity are at particular risk of experiencing recurrent falls during ambulation. It is reasonable that a higher exposure to gait instability during preserved daily activity behavior might influence the risk for frequent falling in a supraordinate way. This risk presumably not decreases before advanced disease stages that are linked to considerably reduced levels of daily activity [17]. However, a clinical advice to reduce ambulatory activity to protect patients from recurrent falling would not be appropriate due to the apparent neuroprotective effects of activity in these patients [46, 47]. Rather, a balance must be found between maintaining activity and applying protective measures that specifically minimize the risk of severe fall-related injuries in these patients.

Whether the severity of ataxic symptoms directly influences fall risk in patients with CA is still controversial. A retrospective study in patients with spinocerebellar ataxias found that fall frequency was associated with the severity of ataxia assessed by the SARA score [4]. However, this association failed to persist in a subsequent prospective follow-up study on the same cohort [5]. A more recent study including patients with sporadic and hereditary forms of CA found no evidence for an association between the SARA score and patients’ fall risk [11]. Our findings support the latter observations but further indicated that the severity of ataxic symptoms becomes relevant for estimating patients’ risk of injurious falling. Accordingly, an increase in the SARA score (subscore for limb ataxia) more than doubled the risk of experiencing fall-related injuries that necessitated medical attention. This association presumably points to a link between the severity of ataxic symptoms (particularly limb dysmetria) and the patients’ capacity for protective postural coping strategies to counterbalance falls. A severe discoordination of limb movements might thus interfere with and hinder the execution of protective postural adjustments while falling.

Taken together, the differential contributions from clinical and instrument-based measures for predicting fall risk in CA encourage and provide guidelines for a multi-level, stepwise fall risk assessment approach in these patients: Accordingly, basic index information that is readily available from medical history taking allows a good estimation of the general fall risk and may promptly inform the clinician which patient would or would not benefit from a more in-depth examination. For those patients at risk of falling, a more elaborate disease severity rating and measures from instrument-based gait and mobility examination provide additional, unique information with respect to the severity of their fall susceptibility and the likelihood for the occurrence of severe fall-related injuries.

### Study Limitations

The cross-sectional study design yielded a heterogenous sample of patients including hereditary, sporadic, and secondary forms of CA which comfounds a direct comparison to previous reports that only considered hereditary forms of CA [4, 5, 18]. However, despite this heterogeneity and the preponderance of earlier disease stages in our cohort, we found a good agreement with existing literature regarding the epidemiology, circumstances, and mechanism of falling in CA. Furthermore, we did not analyze effects of medications on fall risk in our patients with CA. Medication status, in particular, the commonly described “fall risk increasing drugs” such as hypnotics, antipsychotics, antidepressants have been shown relevant risk factors for fall occurrence in geriatric populations [48] and should be considered in follow-up studies. Finally, we are aware that the applied technology for in- and off-laboratory assessment of gait and mobility function is elaborate and currently restricted to specialized clinical centers. However, relevant outcome measures of these assessments (e.g., gait variability or intensity of daily activities) have been shown to be reliably transferable to low-cost wearable technology [15, 49] that could in future facilitate a broad application of a multimodal fall risk screening in patients with CA.

## Data Availability

Data reported in this article will be shared with any appropriately qualified investigator on request after pseudonymization.
